# Long-Distance Counter Calling in Maned Wolves: Friends or Foes?

**DOI:** 10.3390/ani12091081

**Published:** 2022-04-21

**Authors:** Luane S. Ferreira, Victor Sábato, Thiago A. Pinheiro, Edvaldo Neto, Luciana H. Rocha, Júlio Baumgarten, Flávio H. Rodrigues, Renata S. Sousa-Lima

**Affiliations:** 1 Laboratory of Bioacoustics, Department of Physiology & Behavior, Universidade Federal do Rio Grande do Norte, Natal 59078-970, RN, Brazil; fsluane@gmail.com (L.S.F.); victorsabato@gmail.com (V.S.); thiago4jazz@gmail.com (T.A.P.); nettoBlack@gmail.com (E.N.); lua.lupin@gmail.com (L.H.R.); sousalima.renata@gmail.com (R.S.S.-L.); 2 Applied Ecology and Conservation Lab, Universidade Estadual de Santa Cruz, Ilhéus 45662-900, BA, Brazil; 3 Departamento de Biologia Geral, Universidade Federal de Minas Gerais, Belo Horizonte 31270-901, MG, Brazil; rodriguesfhg@gmail.com

**Keywords:** *Chrysocyon brachyurus*, canid, vocal interaction, acoustic parameters, roar-bark, captivity, free ranging

## Abstract

**Simple Summary:**

Maned wolves generally maintain long distances between individuals and are difficult to see in the wild. To understand how they interact, we recorded sequences of alternating long-distance calls (roar-barks) of maned wolves in captivity and in the wild. In natural habitat recordings, we detected more interactions during the mating and initial parental care periods, suggesting communication among mated pairs and, later, among parental caregivers. In captivity, almost all vocal interactions involved both sexes and males presented longer roar-barks compared to females. We measured the same parameter in wild maned wolves and found that the participants in such vocal interactions differ with respect to the duration of their calls, suggesting that maned wolves engaging in long-distance counter-calling are mates. Such acoustic-based inferences of wildlife behavior are cost-effective and can be a useful tool in the conservation efforts to protect this vulnerable canid, and potentially other endangered species that are difficult to observe in the wild.

**Abstract:**

Maned wolves (*Chrysocyon brachyurus*) are monogamous and display biparental care for their young, although adults rarely spend time in close proximity. To better understand vocal interactions of maned wolves over long-distances, we passively recorded >10 months of audio data in the species’ natural habitat and analyzed manual recordings of captive animals, covering the reproductive and non-reproductive seasons. In the natural habitat recordings, we found that maned wolves engage in vocal exchanges (termed interactive sequences) more often during the mating season, suggesting the existence of a partner attraction/reunion/guarding function, and also during the initial parental care period, suggesting communication among caregivers. We analyzed 21 interactive sequences, which were the only instances in which we could distinguish individuals, and found that the individuals interacting differed significantly in their roar-bark parameters, including duration, which also differed between males and females in captivity (male vocalizations were, on average, 0.124 s longer). We also found that interactive sequences in captive animals, involving two or more participants, almost always involved both sexes. These results suggest that acoustic interacting maned wolves are most likely male–female dyads.

## 1. Introduction

The maned wolf (*Chrysocyon brachyurus*, Illiger 1815) is the only large canid from South America (70–90 cm shoulder height, 20–30 kg weight [1]). It is an omnivorous and mainly solitary animal that inhabits savanna-like environments [2]. Due to its low densities (1–8/100 km^2^ [3]), nocturnal–crepuscular habits, and shy nature, the species is hard to study in the wild [4]. Exploring the long-range acoustic communication of cryptic species is an interesting monitoring alternative [5] that has already proven efficient for studying maned wolves [6,7].

Maned wolves produce an explosive roar-bark [8], similar to a longer bark from a domestic dog, emitted in sequences of 5–15 units separated by relatively long intervals of 2–6 s [7]. Roar-bark sequences can often travel more than 1 km, potentially over 3 km [9], making this species more easily detected acoustically than visually [10] (and LSF personal observation). Nonetheless, maned wolves are not highly vocal. In captivity, individuals emit 0.68 roar-bark sequences by night in the reproductive season and 0.28 sequences by night outside the reproductive season [11]. It is hypothesized that this vocalization is used for territorial announcement and defense, mediating same sex spacing [8,11,12].

Maned wolves are monogamous, and pairs use and defend the same home-range, patrolling it separately [13,14]. The species shows biparental care and yearlings may also participate as helpers [4,15]. Therefore, long-range calling could potentially function in intra-familiar group communication [10,16,17].

Contrary to other indirect signs of a species’ presence (e.g., footprints and urine/scat), vocal signs allow the study of real-time interactions between individuals [18], a potential that has been little explored in the maned wolf. Although most roar-bark sequences are emitted by a single individual [8,10,19], 12–33% of sequences involve roar-bark alternations between two or, more rarely, three or more maned wolves (referred to here as interactive, answered, or group sequences [8,10,19]). During intervals between roar-barks, wolves seem to wait and aurally attend to answers [10], suggesting that the long internote intervals may function to facilitate those long-distance vocal exchanges [20].

Pair-mates exchange roar-barks both in captivity [17,19] and in the wild [10,15,17]. Maned wolves may also visually search for their partner after roar-barking [16] and, during estrous, they emit roar-barks whenever the partner is out of visual range [15]. Often partners reunite after the emission of roar-barks or the receiver moves towards the caller [10,19]. Therefore, some authors propose that group vocalizations are primarily pair-mate communication, at least during the breeding season [7,10,19]. Contrary to that, others [12] have found that roar-bark sequences are more often answered by same sex individuals. Free ranging wolves with adjacent home-ranges have been heard exchanging roar-barks and calling toward perceived threats from conspecifics or humans [19]. Maned wolves vocally responded to male and female roar-bark playbacks, although elicited sequences did not alternate with played back roar-barks [9]. These last three pieces of evidence are in accordance with the suggestion that group sequences of roar-barks mediate resource disputes. The majority of the studies mentioned were conducted in captivity, or short opportunistic observations and thus, long term acoustic monitoring should help elucidate maned wolves’ long-distance acoustic interactions.

A previous study exploring our long-term acoustic dataset [7] shows that interactive roar-bark sequences follow the seasonal (but not the lunar or nightly) general pattern of solo/unanswered sequences. However, at that time, recordings during the non-reproductive season were not available. Here we were interested in determining who are the individuals interacting vocally and we predict that if interactive sequences are reproductive male and female communication, then they would be more common during the reproductive season, or some of its periods (mating: March–April, gestation: May; parturition: June; initial parental care: July–September), than during the non-reproductive season. Different rates of interactive sequences depending on the period of the year would also be observed if interactive sequences are disputes for seasonal reproduction-related resources, e.g., territorial disputes may be more intense in critical periods, such as mating and lactation. In this case, we would expect same sex vocal interactions (disputing for mates, similar to jackals [21]) or no sex bias in individuals participating in roar-bark exchanges (interfamilial resource disputes). If interacting maned wolves are male and female dyads, then the animals’ difference in sexually dimorphic acoustic parameters will be large (significantly different from zero). Finally, if interactive sequences are mostly emitted in one of the aforementioned contexts, then we should find the same trend in captivity data, where we have the identity of participants.

## 2. Materials and Methods

### 2.1. Natural Habitat Area

**Figure 1:**
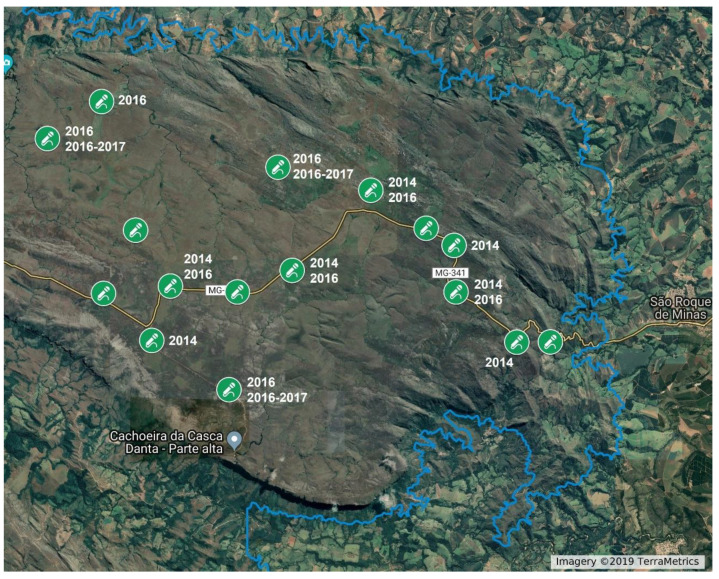
The location of acoustic recorders at Serra da Canastra National Park in Minas Gerais state, Brazil. Microphone symbols indicate the position of the autonomous recorders (Wildlife Acoustics SongMeters SM2+). Recorders with no noted years were active in 2014 (April–July), 2016 (March–June) and 2016–2017 (December–January) periods. The blue line indicates the park boundaries. Image from Google Earth © 2019 TerraMetrics.

Recordings were done at the Serra da Canastra National Park, in Minas Gerais state, Brazil (Figure 1). The Park is mainly composed of highland Cerrado open savannas with a cold, dry season (April–September) and a hot, rainy season (October–March) [22]. Previous capture-recapture studies indicate a density of 8 maned wolves per 100 km^2^ (considered high [23]), with home ranges averaging 80 km^2^ [13].

### 2.2. Natural Habitat Data

All natural habitat recordings were made passively, with autonomous recorders SongMeter SM2+ (Wildlife Acoustics, Inc., Concord, MA, USA) coupled with a single SMX-II omnidirectional weatherproof microphone each (Wildlife Acoustics, Inc., Concord, MA, USA). Recorders were distributed broadly in high places (1373.0 ± 56.6 m altitude) and attached to 1.4 m wooden stakes. The equipment was set to record with +36 dB gain, 8 kHz sample rate, and 16-bit wave format coding.

In 2014 we recorded between 5 April and 8 August, during the maned wolf reproductive season. We deployed 12 autonomous recorders (Figure 1, “2014” labels) set to record from 18:00 p.m. to 6:00 a.m. every 24 h. In 2016 we recorded from 9 March to 1 July, also during the maned wolf reproductive season. We deployed 13 autonomous recorders (Figure 1, “2016” labels) set to record from 17:00 p.m. to 5:00 a.m. every 24 h. Between 1 December 2016 and 31 January 2017, during the maned wolf non-reproductive season, we deployed 8 autonomous recorders (Figure 1, “2016–2017” labels) programmed to record during the first 3 h of the night (17:45–20:45 p.m.: the period of highest maned wolf roar-bark activity [6]). The non-reproductive recording scheme differed from the previous ones because it was used for a multi-objective project.

Equipment malfunction, due to low battery and equipment wear, restricted the use of all the data. Nights during which at least half of the deployed recorders were active were considered for analyses. During 2014, 118 nights were considered with 11.9 ± 0.3 (mean ± SD) average active recorders, in 2016 we considered data from 105 nights with 12.3 ± 1.8 average active recorders and, in 2016–2017, we considered 37 nights with 6.3 ± 1.2 average active recorders. Since the number of recorders and the times recorded differed among years, all sequence counts were divided by the number of active recorders, and only the first 3 h of the night were selected for subsequent analyses. That is, between 17:45 p.m. and 20:45 p.m. in 2016 and 2016–2017 and from 18:00 p.m. to 21:00 p.m. in 2014.

Some areas recorded could be more intensely used by maned wolves than others, which would influence the probability of detecting their roar-barks. As the recorders’ sites varied between years (Figure 1), and not all of them were active during the same nights, the analysis with the full dataset could be spatially biased/unbalanced. In an effort to control for that, we made a subsampled data including only the 5 recording sites that were used in all years (sites with no label in Figure 1), and only nights in which all of those 5 recorders were active. We analyzed both the data with all recorders and the subsampled with only 5.

### 2.3. Roar-Bark Sequence Detection, Counting and Classification

Roar-barks were detected in the audio files using an automatic detector built in XBAT-R7 (Extensible Bioacoustic Tool [24]) extension for Matlab R2011a (MathWorks, Inc., Natick, MA, USA). Detections were manually validated according to a published protocol [25]. The end of a sequence was arbitrarily defined as the point when there was more than 10 s between any roar-barks, independent of the emitter [6]. This definition is important for moments of high vocal activity.

**Figure 2:**
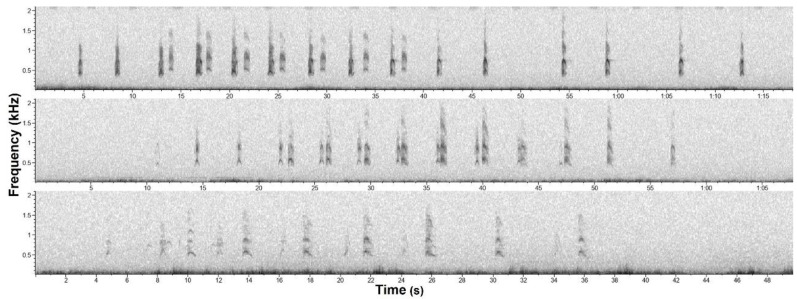
Three examples of maned wolf interactive roar-bark sequences recorded passively at the Serra da Canastra National Park, MG/BR. Each was registered in a different recorder site, on 19 July 2014 at 1:55 a.m., 9 April 2016 at 19:21 p.m., and 14 March 2016 at 20:00 p.m., respectively from top to bottom. The audio files of those examples are in Supplementary Material Audio S1–S3.

For each sequence found we noted the date, begin time, and the number of animals involved. Multiple participating maned wolves can be detected by differences in cadence, relative intensity, time of arrival at different sensors, and spectral characteristics of roar-barks, as well as occasional overlaps which indicate calls came from different individuals (Figure 2; Supplementary Material Audio S1–S3). As the recordings were made passively, that is, with autonomous recorders and no visual information, interactive roar-bark sequences are the only instances we can distinguish between free ranging individuals. Although determining which individual emitted which roar-bark is usually easy when there are only two animals, sequences involving more than two jeopardizes individual discrimination of roar-barks, as the calls signal-to-noise ratio is commonly too low to allow any comparison.

We classified sequences as “solo” when there was no indication of more than one animal participating, or “interactive” when there was more than one maned wolf alternating roar-barks. Sequences in which the presence of another animal was uncertain (e.g., a solo sequence with one faint mark in between that may or may not be a roar-bark) were not included in either category. When those categories are not mentioned the data refers to all vocal activity, combining solo, interactive, and uncertain sequences. We also use the term “dyad sequence” to refer to any interactive sequence involving only 2 animals. For the natural recordings the term is almost a synonym of interactive sequences, as more than 2 animals in the same sequence is very rare in our wild dataset. However, the term is very useful for the captivity dataset.

We divided our sample in parts within the reproductive cycle of the species that we refer to as ‘periods’. We considered the mating period from 1 March to 20 April and the gestational period from 21 April to 31 May (shortened because the exact time of conception and parturition varies). The parturition period was considered to be June and the initial parental care was in July (our latest records during the reproductive season). These periods are based on the reported mating period for the species [15], a gestation of 65 days [26], and the reported peak in births for the Serra da Canastra National Park in June [4,19,27]. We have confirmation that at least one female in the area was lactating in July 2014 and 2016 (R. C. de Paula, personal communication). The 2016–2017 dataset was considered as the non-reproductive period.

Besides calculating nightly sequences by recorder and nightly percentage of interactive sequences, we made 4 comparisons between periods: the proportion of solo/interactive sequences (number of roar-bark sequences), the proportion of nights with interactive sequences versus with solo sequences only (nights without vocal activity are not included), the proportion of nights with versus without sequences (nights with solo sequences only and without vocal activity are both included in the last category), and the proportion of all four categories of nights (without vocal activity, with solo sequences only, with interactive sequences only, and with any kind of vocal activity).

### 2.4. Relative Distance Estimation

**Figure 3:**
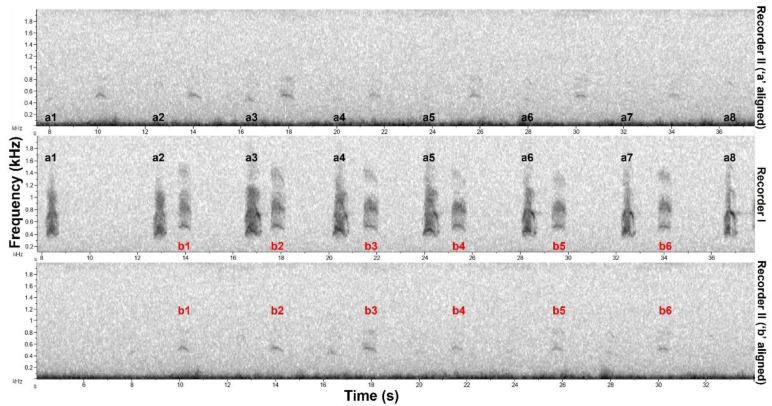
Interactive roar-bark sequence with two participating maned wolves (a and b). This sequence was registered in two recorders (I and II). Roar-barks (numbers) only align in recorder II one animal at a time (top and bottom) because individuals are at different positions. The spectrogram shown in recorder I (middle) is a portion of the first roar-bark sequence example in Figure 2.

Sometimes the same sequence was registered by more than one autonomous recorder (Figure 3). As the recorders were not time synchronized, time cannot be used to guarantee it was the same sequence. Certainty about it stems from several idiosyncrasies in maned wolf roar-barks sequences, e.g., unique inter-roar-bark intervals. This way, if we can temporally align roar-barks recorded in two or more different recorders, it becomes easy to evaluate if it is the same sequence. To avoid counting sequences twice in the seasonal analysis we only considered the recording with the highest signal-to-noise ratio. In some rare cases an interactive sequence was registered by more than one recorder. In most of those cases the alignment of roar-barks between recorders was only possible with the roar-barks of a single animal at a time (Figure 2). This happens because the acoustically interacting maned wolves are not at the same position [7]. Therefore roar-barks of each animal will travel different distances to reach each recorder and will arrive at different times in relation to each other.

We used the time of arrival (TOA) difference between the roar-barks of the different animals in each recorder to make estimates of the relative distance between vocalizing wolves. If animals are vocalizing together, the TOA difference will be zero and as animals are further away from each other it will increase. For instance, in Figure 2 the time difference could be calculated by the time between a3 and b3 in recorder I minus the time between the same roar-barks in recorder II. Considering a sound speed of 343 m/s and that the time difference is created in both recorders, we used the formula “(time difference/2) × 343” to calculate a distance in meters. We recalculated this estimation for all interactive sequences registered by multiple recorders that had enough quality for the time difference measure. We used any interactive sequence that met these criteria, independent of hour or time of the year.

### 2.5. Captivity Data

**Figure 4:**
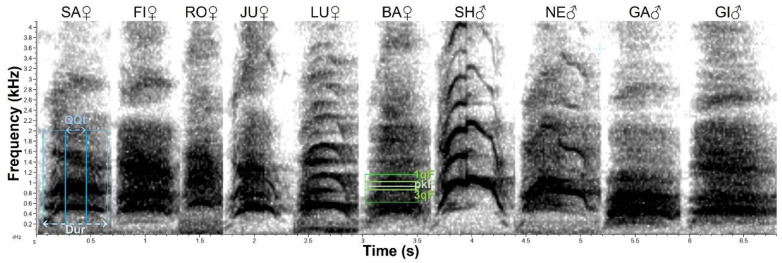
Roar-barks of captive maned wolves. The first box (blue) exemplified in SA shows the entire selection (200–2000 Hz), in which the Total Duration (“Dur” in the image) and InterQuartile Duration (“QQt”) are measured. The second box (green) exemplified in BA shows the selection of the second frequency band, in which the 1st Frequency Quartile (“1qF”), the Peak Frequency (“pkF”), and the 3rd Frequency Quartile (“3qF”) are measured. Spectrogram made in Raven Pro. 1.6.

We used data collected in 2011 [11] at two facilities in the state of Minas Gerais (Brazil): the Criadouro Científico de Fauna Silvestre para Fins de Conservação da Companhia Brasileira de Metalurgia e Mineração (CC-CBMM) and the Zoológico da Associação Esportiva e Recreativa dos Funcionários das Usinas Siderúrgicas de Minas Gerais (ZOO-USMG). Animals were recorded with a unidirectional microphone, Sennheiser K6-module ME-66 (40–20,000 Hz ± 2.5 dB flat response frequency), connected to a Marantz PMD-661 solid state recorder using 96 kHz sampling rate and 24-bit wav encoding format. At CC-CBMM, acoustic monitoring was done for 40 nights during the breeding season (between April and June) sampling calls from four adult captive maned wolves females (SA, FI, JU, RO) and 2 males (SH, NE). Two pairs were housed together (SH + SA, NE + FI) and the remaining females were housed separately in enclosures with no other wolves. RO is the mother of NE and JU which have different fathers, and JU is the mother of SA. The males that sired the captive animals were no longer in the facility. At ZOO-USMG the acoustic monitoring was done for 20 nights outside the breeding season (November). Four adult captive maned wolves were recorded. They were housed in two pairs (male + female: GA + LU and GI + BA). The males are litter siblings. An example of each captive maned wolf roar-bark is shown in Figure 4. Data from captive animals was used to determine sexually dimorphic acoustic parameters. We additionally counted the number of solo and interactive roar-bark sequences recorded in each season, as well as the sex and identity of captive participants.

### 2.6. Acoustic Parameters

We looked for acoustic parameters that were robust against the effects of propagation through distance, or that were little affected relative to the variation due to individual differences (in mammals [28,29]; in maned wolves [20,30]). Parameters measured in the roar-barks were taken automatically after each call was manually selected in Raven Pro 1.6 (Bioacoustics Research Program, 2014. Ithaca, NY, USA: The Cornell Lab of Ornithology; http://www.birds.cornell.edu/raven, accessed on 13 January 2022).

The acoustic parameters selected were (Figure 4): the roar-bark Total Duration, in seconds; the InterQuartile Duration, in seconds (the duration between the moment of 25% energy accumulation in time and the moment of 75% energy accumulation in time); the 2nd Band Peak Frequency, in Hertz (the frequency of highest intensity of the second frequency band, which is usually at 600–1000 Hz); the 2nd Band 1st Frequency Quartile, in Hertz (the frequency that accumulates 25% energy of the second band); and the 2nd Band 3rd Frequency Quartile, in Hertz (the frequency that accumulates 75% energy of the second band). The parameters descriptions were taken from Raven Pro User’s Manual [31].

The acoustic parameters of the captivity dataset were measured in spectrograms built in Raven Pro 1.6, using Hann window, 4096 window size, 50% overlap, 50% brightness, and 75% contrast. For the natural habitat recordings, we used spectrograms with 512 window size, 45–55% brightness, and 60% contrast. One selection box was made comprising the first two frequency bands of roar-barks, fixed from 200 Hz to 2000 Hz (Figure 3 blue box), for the extraction of the Total Duration and the InterQuartile Duration. A second selection box was made comprising only the roar-bark second frequency band (Figure 3 green box), around 620 Hz to 1000 Hz depending on the roar-bark, for the extraction of the remaining three frequency parameters.

Twenty good quality roar-barks (high signal-to-noise ratio) of each captive individual were used (total: 200 roar-barks). We tried to include roar-barks of as many different sequences as possible, but some animals emitted very few of them. The number of sequences from which we selected roar-barks varied from 3 to 19 per individual, totaling 56 distinct sequences, of which 23 were solo and 33 interactive sequences (in some of which, we selected calls from more than one wolf). To control for those factors, we noted for each captivity roar-bark the emitting individual, the sequence it belonged to and how many participants the sequence had.

In the recordings from the natural habitat, we used any good quality interactive sequence, independent of hour or time of the year. We selected interactive sequences in which both animals had at least 3 good-quality and confidently identified (as belonging to the same animal) roar-barks each. Our goal was 5 good quality roar-barks, but that was not always possible. We averaged the acoustic parameters’ values from roar-barks of the same animal in a sequence in order to avoid pseudoreplication, and also to stabilize parameters possibly affected by varying noise characteristics (wind, other zoophony, etc.). Pairwise comparisons were conducted, that is, each animal was only compared to the one it was vocally interacting with in the interactive sequence considered. We tried to select homogeneously distributed interactive sequences across all periods, registered at different sites (recorders) and avoided sequences emitted less than a night apart from each other. This was done to avoid biasing the data with many sequences from the same individuals. In total, 21 interactive sequences from the natural habitat recordings were selected for analyses.

To visually show the distribution (in box plots) of the roar-bark parameters in the natural recordings, we classified them based on which part of the spectrum they occupy: for each interaction the wolf with the smaller value of “duration” between the 2 was assigned to the “shortest” set, and the other to the “longest”; the wolf with the smaller value of “frequency” between the 2 was assigned to the “lowest” set, and the other to the “highest”.

### 2.7. Statistical Analysis

Data was generally not normally distributed (tested with Shapiro–Wilk normality test), so we used Kruskal–Wallis tests, followed by pairwise Wilcoxon tests adjusted by the Benjamini–Hochberg method [32], for the seasonal analysis. To compare the seasonal variance in proportions of sequences and of night composition we used chi-square tests, followed by Fisher’s exact test if any category had less than 5 counts, and a comparison of Pearson’s residuals to find the groups and categories that deviated from the expected proportion. To compare captive male and female acoustic parameters we transformed the non-normal parameters using the Yeo-Johnson method [33] and then fitted linear mixed models to each parameter controlling for the random factor sequence nested in individual. We also included in the fixed factors the number of participants (one/two/three or more) to evaluate if the sequence type influences the acoustic parameter. The final formula was acoustic parameter ~sex + participants + (1|individual/sequence). We used ANOVAs followed by Tukey contrasts to test the effect of fixed factors and their levels. To test the difference in the acoustic parameters of participants of interactive sequences in the natural habitat recordings we used paired Wilcoxon tests. All statistics were computed in R (R version 4.1.1 (10 August 2021)—“Kick Things” Copyright © 2021 The R Foundation for Statistical Computing, Vienna, Austria).

## 3. Results

### 3.1. Seasonal Distribution of Roar-Bark Sequences: When They Interact

Considering only the first 3 h of the night, we found a total of 523 maned wolf roar-bark sequences in the combined datasets; of those, 58 were interactive sequences (11%). Only two interactive sequences showed engagement of three or more animals instead of two.

**Figure 5:**
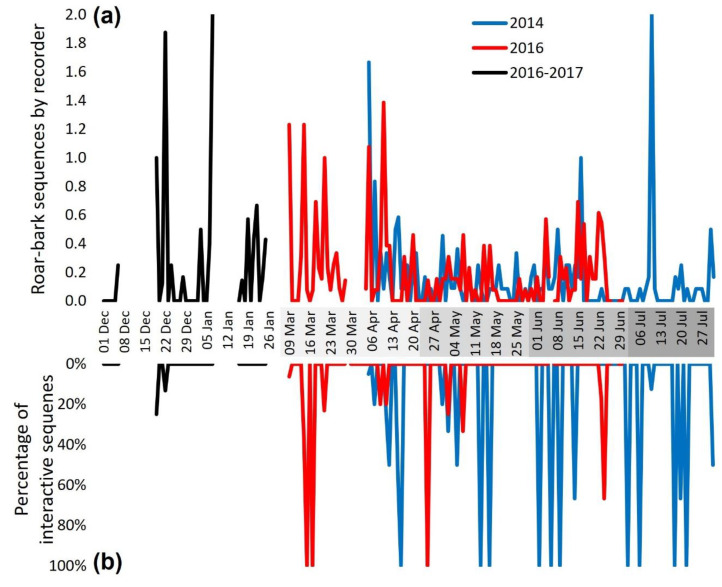
(**a**) Seasonal distribution of maned wolf roar-bark sequences recorded at the Serra da Canastra National Park, Brazil. Each point is a night (the first 3 h). Periods are separated by the grayscale in the dates (non-reproductive, mating, gestation, parturition, and initial parental care, consecutively). The number of sequences found was divided by the number of active recorders at that moment (4–13 Wildlife Acoustic SongMeters 2). (**b**) Percentage of sequences in which 2 maned wolves were participating.

The distribution pattern during the reproductive season (Figure 5a) shows a high vocal activity during the mating period, a decrease during the gestational period, and a smaller increase circa parturition (with an occasional peak in the initial parental care period). The number of interactive sequences in general followed the vocal activity pattern, except for the non-reproductive season that presented only four interactive sequences in two nights (Table 1). There were many nights with high percentages of interactive sequences in the reproductive season, especially in March and from June on (Figure 5b).

Unexpectedly, the vocal activity during the non-reproductive season was as high as in the mating period (Figure 5a). However, visually the occurrence of interactive sequences was rare during this period (Figure 5b).

**Figure 6:**
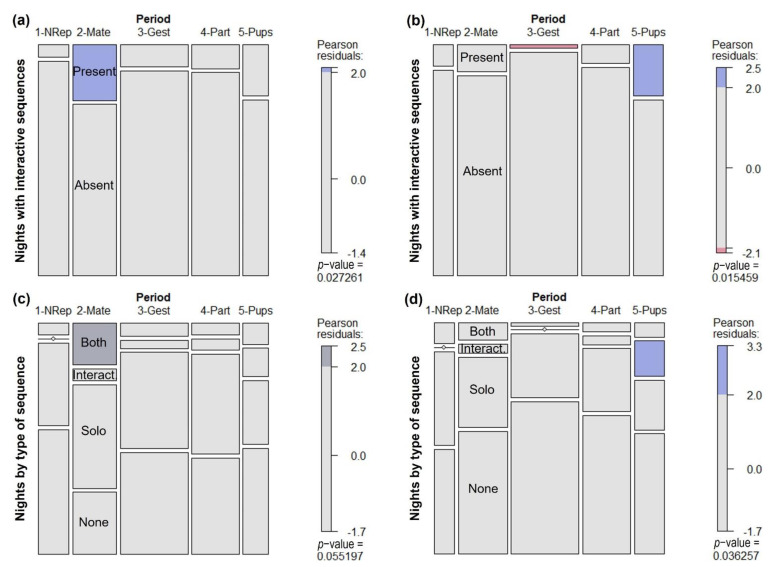
Mosaic plots showing the proportion of nights in each roar-bark activity category through time. Each mosaic tile area is proportional to the observed number of nights in each category (height) and the number of nights sampled in each time period (width). Time periods are as follows: NRep = non-reproductive; Mate = mating; Gest = gestation; Part = parturition; and Pups = initial parental care. Pearson residuals were used in a Chi-Square Test of Independence to verify if there is a difference among observed and expected values. Blue tiles have significantly greater frequency values than expected, dark grey tiles have greater frequency values than expected, but marginally significant, and red tiles have significantly smaller frequency values than expected. All other tiles show no significant difference among observed and expected values. (**a**) Proportions of nights with and without interactive roar-bark sequences using the entire dataset; (**b**) Proportions of nights with and without interactive roar-bark sequences using the subsampled dataset considering only the 5 recorders common to all periods; (**c**) Proportion of each category of vocal activity using the entire dataset; (**d**) Proportions of each vocal activity category using the subsampled dataset considering only the 5 recorders common to all periods. Categories of vocal activity are as follows: None = no vocal activity; Solo = only solo roar-bark sequences; Interact. = only interactive roar-bark sequences; and Both = solo and interactive sequences. Plots made in R with function mosaic {vcd}.

Considering the entire periods, for the complete dataset, the initial parental care presented a tendency for a higher proportion of interactive sequences compared to the expected solo/interactive sequence proportion (Chi-squared test = 8.823, df = 4, *p* = 0.0657). The mating period presented a higher proportion of nights with interactive sequences compared to the expected proportion of nights with and without any interactive sequence (Figure 6a; X^2^ = 10.939, df = 4, *p* = 0.0273, Fisher’s exact test: *p* = 0.0329) and a tendency of a higher proportion of nights with both solo and interactive sequences compared to the expected proportion of night composition (no sequence, solo only, interactive only, both; Figure 6c; X^2^ = 20.684, df = 12, *p* = 0.0552, Fisher’s exact test: simulated *p* = 0.0705).

For the dataset including only the five recorders in common, the initial parental care period presented a higher proportion of interactive sequences compared to the expected solo/interactive sequence proportion (X^2^ = 20.003, df = 4, *p* = 0.0005, Fisher’s exact test: *p* = 0.0019), a higher proportion of nights with interactive sequences compared to the expected proportion of nights with interactive sequences and with only solo sequences (X^2^ = 10.003, df = 4, *p* = 0.0404, Fisher’s exact test: *p* = 0.0352), a higher proportion of nights with interactive sequences compared to the expected proportion of nights with and without any interactive sequence (Figure 6b; X^2^ = 12.269, df = 4, *p* = 0.0155, Fisher’s exact test: *p* = 0.0083), and a higher proportion of nights with interactive sequences only compared to the expected proportion of night composition (Figure 6d; X^2^ = 22.114, df = 12, *p* = 0.0363, Fisher’s exact test: simulated *p* = 0.0485). In addition, the gestational period presented a smaller proportion of nights with interactive sequences compared to the expected proportion of nights with and without any interactive sequence (Figure 6b; X^2^ = 12.269, df = 4, *p* = 0.0155, Fisher’s exact test: *p* = 0.0083).

**Figure 7:**
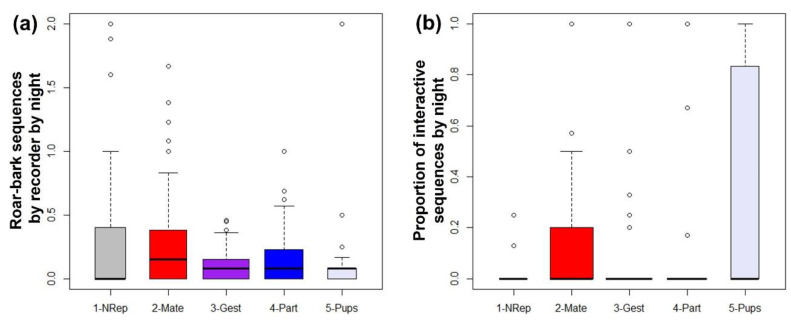
(**a**) Number of maned wolf roar-bark sequences by recorder by night; and (**b**) Proportion of interactive sequences by night during each time period. Time periods are as follows: NRep = non-reproductive; Mate = mating; Gest = gestation; Part = parturition; and Pups = initial parental care. Boxes’ widths are proportional to the number of nights sampled.

Figure 7a shows the sequences by night pooled together by period. The Kruskal–Wallis test (Kruskal–Wallis chi-squared = 12.778, df = 4, *p* = 0.0124), followed by pairwise Wilcoxon comparisons, indicated that during the mating period there were significantly more sequences by recorder by night than in the gestational (BH adjusted *p* = 0.0097 Mate × Gest) and the initial parental care period (*p* = 0.0215 Mate x Pups). The mating period was not higher in vocal activity than the non-reproductive and the parturition period (*p* > 0.05). Despite the visual impressions of Figure 7b, the nights with sequences (any) did not differ significantly in their proportion of interactive sequences in the night across periods (K-W X^2^ = 8.1282, df = 4, *p* = 0.08699).

The subsampled data with only the five common recorders presented no significant difference in the number of sequences by night between periods (K-W X^2^ = 7.4627, df = 4, *p* = 0.1134), but the proportion of interactive sequences by night was significantly greater in the initial parental care period than in the gestational period (K-W X^2^ = 11.318, df = 4, *p* = 0.0232; *p* = 0.0170 Gest × Pups).

In the comparative Table 1 it is possible to see that, on average, the percentage of interactive sequences by night and the percentage of nights with interactive sequences was low in the non-reproductive and gestational periods. The average interactive sequences by night (by recorder in the full sample) is high in the mating and parental care periods, and particularly low in the gestational period.

### 3.2. Distance Estimation: How Close to Each Other They Interact?

**Figure 8:**
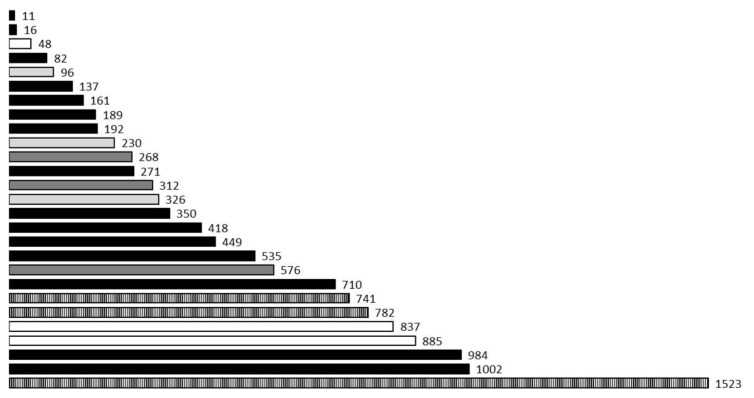
Estimated distance (in meters) between maned wolves exchanging roar-barks in sequences recorded simultaneously in 2 recorders. Non-black bars represent cases (4) where the same interactive sequence was recorded with enough quality in 3 recorders, and thus 3 different distances were calculated for each of them (using the time difference between recorders A × B, B × C, and A × C.).

We were able to estimate the distance between participating maned wolves in 19 interactive roar-bark sequences. In four of those cases the sequence was registered with enough quality in three recorders (Figure 8: non-black bars). For those we could calculate three distances (A–B, A–C, B–C) instead of just one. Those four cases were consecutive interactive sequences from 01:19 a.m. to 02:05 a.m. of 19 July 2014 (one of them is the example in Figure 3). The estimated distance between animals decreases along these four interactions (1523 m, 885 m, 576 m, 326 m: larger of the three distances), suggesting animals walked to meet each other (approximately 0.43 m/s if only one moved).

Including all relative distance values calculated (*N* = 27), the estimated distance between individuals was 449.32 ± 374.36 m (mean ± SD). The two smallest values were 11 m and 16 m and the largest 1523 m. The shortest distance interactions could be considered as animals together since the estimation is not precise and those distances allow visual contact among individuals. Note that distances calculated for the same interactive sequence varied in the values of estimated distance (e.g., 885 m for recorders A–B and 48 m for recorders B–C). This happens because the estimated distance is relative to the axis formed by the aligned recorders used for the calculation. Therefore, any of those distances may not be the true maximum linear distance between animals, although they should be close to the minimum.

### 3.3. Captivity Dataset: Who Is Interacting in Captivity?

There was a total of 89 recorded roar-bark sequences at the captivity facility during the breeding season (CC-CBMM: four females, two males). Of those, 60.7% were solo sequences, 41 emitted by males, 10 by females, and 3 by unidentified maned wolves from areas outside the facility. A proportion of 39.3% of those were interactive sequences, 15.7% dyad sequences and the remaining 23.6% involving three or more animals.

Thirteen out of fourteen dyad sequences involved a male and a female. The remaining dyad sequence was an interaction between mother and daughter (JU/SA, adults, not housed together). The random chance of any dyad sequence being composed of a male and a female was 53.3%. Nine dyad sequences were made by the same male/female (SH/JU, that were not housed together) and three by this male and another female (2 with FI and 1 with RO, neither of these females housed with SH). The female housed with SH (SA) was the least vocal animal, normally only roar-barking when many maned wolves were engaged in long distance calling. The only dyad sequence the other male (NE) made was with female FI, his enclosure mate. SH and JU were the most vocally active captive maned wolves, participating in 71 (39 solos/12 dyad sequences/20 3+ individuals) and 30 (3/10/17) sequences respectively. FI was the third most vocal animal, participating in 26 sequences (7/4/15).

Considering sequences involving more than two animals (*N* = 21), 18 had the participation of both males (SH and NE) and two of just SH. Female JU was engaged in 17 out of those 21 sequences, female FI in 16, and in 12, both females were participating. The participant composition of the remaining sequences with more than two animals varied. Except for the JU/SA dyad sequence, all interactive sequences had the participation of both sexes.

At the facility recorded outside the breeding season (ZOO-USMG: two females, two males) only 10 roar-bark sequences were recorded, three were solo sequences (two emitted by male GA and one by female LU). All dyad sequences (*N* = 4) involved a male and a female, two being made by GA/LU (enclosure mates), one by GA/BA, and one by GI/BA (enclosure mates). The chance that a random dyad sequence would involve a male and a female was 2/3 in this case. The remaining three sequences were made by GA/GI/BA.

### 3.4. Acoustic Parameters: How Do Participants of Interactions Differ?

Total Duration was the only normally distributed parameter (Shapiro–Wilk W = 0.9923, *p* = 0.3748), and therefore not transformed, and the only whose model is in Table 2. The linear mixed model of the captivity roar-barks Total Duration revealed a significant effect of factor sex when controlling for individual and sequence (ANOVA: numDF = 1, denDF = 8, F = 5.8582, *p* = 0.0418). Males were longer than females (Table 2; Tukey contrasts: m-f Z = 2.483, single-step adjusted *p* = 0.015). In no other model was sex a significant factor, although there was a tendency in the model for InterQuartile Duration (ANOVA: numDF = 1, denDF = 8, F = 4.3681, *p* = 0.0700) in the same direction (males on average 0.030 s longer: untransformed values). The type of sequence was not a significant factor in any parameter model, except for the InterQuartile Duration (ANOVA: numDF = 2, denDF = 77, F = 5.1698, *p* = 0.0078). For this model, roar-barks of sequences with three or more participants were slightly longer (Tukey ‘3more’–’one’: Z = 2.808, *p* = 0.0136; ‘3more’–’two’: Z = 2.377, *p* = 0.0454; untransformed average difference of 0.020 s from ‘3more’ to ’one’). Individuals were responsible for a considerable variation in the time parameters (see Table 2 for Total Duration), but almost none in frequency parameters nor the factor sequence in any parameter (all estimates of those random factors are at least four orders of magnitude smaller than the intercept estimate; see Table 2 for Total Duration).

In the natural habitat we could measure the acoustic parameters of 21 interactive roar-bark sequences in the recordings (from 160). Unfortunately, none of the four interactive sequences in the non-reproductive period had enough quality (signal-to-noise ratio) to be used. All other months have at least one sample.

**Figure 9:**
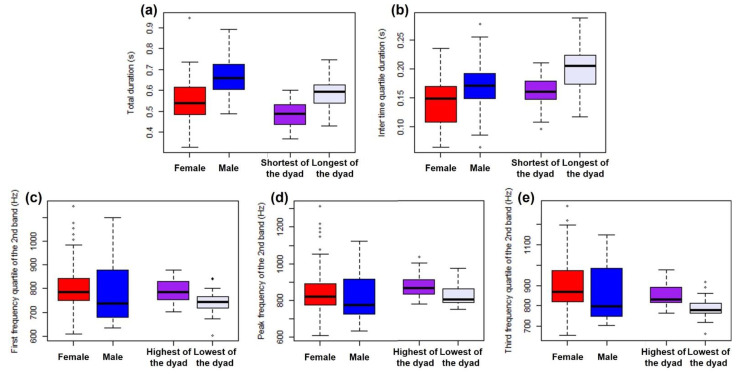
Acoustic parameters of maned wolf roar-barks analyzed separately for captive (recorded manually in 2 facilities at Minas Gerais state, Brazil) and for free ranging wolves (recorded passively in the Serra da Canastra National Park, MG/BR). In captivity the differences between females (red) and males (blue) were tested using a linear mixed model controlling for individual and sequence (males are longer, no other parameters significantly differ). The parameters of free ranging wolves were measured and paired within each dyad interactive roar-bark sequence (2 animals) using paired Wilcoxon tests. Individuals were only compared to the one they were interacting at the moment (participants differ significantly in all parameters). For visualization only, for each comparison the shortest in duration and highest in frequency of the dyad was set in the first box (purple) of the free ranging data and the other in the next box (lavender). (**a**) Total duration; (**b**) InterQuartile duration; (**c**) 1st frequency quartile of the 2nd band; (**d**) Peak frequency of the 2nd band; and (**e**) 3rd frequency quartile of the 2nd band.

In the natural habitat recordings, all five acoustic parameters were significantly different between participants of interactive sequences in the paired Wilcoxon tests (Total Duration: V = 0; InterQuartile Duration: V = 0; Peak Frequency of the 2nd Band: V = 210; 1st Frequency Quartile of the 2nd Band: V = 231; 3rd Frequency Quartile of the 2nd Band: V = 231; all *p* < 0.0001). The comparison of roar-bark parameters of free-ranging animals with those parameters from captive females and males can be seen in Figure 9.

## 4. Discussion

In this work we aimed to determine when and who engages in acoustic interactions in free-ranging maned wolves. Using passive acoustic monitoring we discovered that there were more interactive roar-bark sequences (two animals engaging in vocal exchange) during the mating and initial parental care periods. This finding is in accordance with our prediction that interactive roar-bark sequences mediate intrafamilial group long-distance communication. We also found that participants of these interactive sequences differed in the acoustic parameters of their roar-barks, including their duration, which in captivity differed between males (longer) and females. Additionally, we found that in captivity, almost all roar-bark dyad sequences (two participants) were between a male and a female and interactive sequences with three or more participants always included both sexes. Taken together, our evidence indicates that maned wolves in our free-ranging recordings that are acoustically interacting at long distances are most likely male-female dyads.

Maned wolves usually remain several hundred meters from each other [14], and female estrus lasts only five days [26]. During estrus, long distance vocal exchanges could help receptive individuals to find each other to mate. Our findings suggest that when maned wolves interact vocally, they are far away from each other (mean of 450 m), which supports the function of roar-barks as means of individual location. The evolution of such a strategy can positively influence the maintenance of low-density populations subjected to the Allee effect (positive density dependence in mating probability [34,35]). Mate limitation reduces reproduction when males and females have difficulty finding each other, thus acoustic communication may be crucial for the conservation of the species in the wild. In addition to location, roar-barking whenever the partner vocalizes can serve as a mate guarding strategy. That is, if the first caller is announcing its receptivity, then the second animal could be announcing to third parties that the mate is taken and will be defended.

Besides the mating period, we found many interactive sequences circa parturition and parental care periods. Interestingly, the only interactive sequence registered in a study in the ecological station of Itirapina (SP/Brazil) was on 19 June [36], in accordance with our findings. During these periods, the function of interactive roar-bark sequences could be communication among caregivers. The most reported direct paternal care involves bringing food to the lactating female or offspring [14,16,19,37]. The female’s response to the male calling may help him locate the den, as she may move the pups frequently from den to den [16,19]. When the pups are around 4–5 weeks old they start following the female [16], and therefore the male would still benefit from real-time clues of their positions. Otherwise, the female may be vocally soliciting care. Vocal exchanges between the mated pair may be a form of parental care negotiation or manipulation [38,39]. Future studies investigating which sex initiates these interactive sequences would help clarify this issue.

Lastly, the simultaneous vocalizations, at any period, could be a verification that the other pair member is still alive and present in the territory. If positive, it serves as an announcement to neighbors and potential intruders, similar to one of the proposed functions of avian duet [40,41]. Maned wolves have been observed extending their range [14,37] or invading territories after the death/disappearance of one of the owners of the breeding pair [10,19]. Thus, the vocal presence of both individuals in a mated pair may reinforce their resource-holding potential to individuals looking to establish new territories or expand their boundaries. During the reproductive season (particularly the mating and parental care periods in our case) territorial defense may become more important, and therefore pairs would make more interactive sequences at this period, when (reproductive) stakes are highest. Territorial defense during this last period could also be considered an indirect form of parental care [42].

A small number of the interactive sequences in our natural habitat dataset may involve same sex individuals. Such events have been reported [10,19], especially in captivity [8,12], and suggest that roar-barks are multifunctional signals [6,7]. There are also reports of juveniles from the same home range participating in interactive vocal sequences [10]. Thus, those potential helpers would eventually create same sex vocal exchanges. Furthermore, juveniles probably have an underdeveloped vocal apparatus, which could bias our acoustic parameter analyses. Finally, we could only analyze the acoustic parameters of 21 interactive sequences (of 160 recorded), and none in the non-reproductive season, limiting our ability to conclude anything about sex discrimination in these vocal exchanges. Nevertheless, this is a general problem of studies in natural habitats, and we consider ours very extensive, in area and time recorded.

Our captivity data supports the idea that female–male communication is the main function of long-distance vocal interactions. Except for a mother–daughter dyad sequence, all other dyad sequences (*N* = 17) were female–male dyads. We would expect many more female–female dyad sequences, as the random chance for that was around 37%. The chance for a male–male dyad sequence was lower (7.5%), but as males are more vocally active [11] we would still expect at least one interaction of this kind. It should be noted, though, that most dyad sequences (13/17) were not made by enclosure mates, which apparently does not support the intrapair communication hypothesis. However, one of the alleged benefits of such exchanges stems from relocating mates separated by long distances, and thus, enclosed in a limited area, the need for such an encounter facilitation mechanism is absent. Additionally, captive pairs are mated artificially, not involving natural sexual selection by the animals. Therefore, the lack of same-enclosure dyad sequences could also be the result of weak pair bonds.

Another aspect to consider in captivity is that, contrary to our natural habitat findings, interactive sequences with more than two animals (*N* = 24) were more common than dyad sequences. As calling by one individual stimulates calling in other maned wolves [8,11,12], group sequences may be more common in artificially closer proximity situations. Curiously, sequences with more than two animals always involved both sexes. Hence, our data does not support the idea that maned wolves respond more to same sex individuals (as found by [8,12]). Instead, captive maned wolves could respond more to any opposite sex individual and the composition of interactive sequences may only reflect the individuals’ disposition to vocalize. This last characteristic could be a product of dominance status and/or individual differences (i.e., personalities).

Larger vocal folds and vocal apparatus can produce sounds of longer wavelength [43] and more voluminous lungs may allow to sustain a loud vocalization longer [44]. The last is in agreement with our captivity findings that males produce longer roar-barks and are slightly larger (on average 2 kg and 2.5 cm bigger [14]). Contrary to what we had expected, though, the difference in frequency was not significant and more likely due to individual variance. Maybe the small sexual dimorphism in the species is not enough to produce detectable frequency discrepancies or that differences may only become significant with a larger sample size. This can also mean the difference in duration may be less related to anatomy and instead be related to motivation [11,45,46]. In the context of opposite sex communication, the motivation would be to advertise sexual quality and/or territorial holding potential (for joint territorial announcement). In any of those cases, female and male motivation would be the same and vocalizations would tend to be more similar in duration between sexes. As this is not the case, maybe males are more motivated to attract extra-pair mating or be subject to more intense competition/selection. More studies are needed to elucidate this matter, but it is important to reflect that if the differences are motivational, then it is possible that captivity and natural habitat roar-bark acoustic parameters trends should differ, as motivation is context dependent. This would be a caveat for our assumptions about sex discrimination in free-ranging animals.

## 5. Conclusions

Here we started from passive acoustic recordings with no visual information and ended up with new information on real-time interactions between maned wolves, based on temporal distributions and differences in acoustic parameters in their long-distance calls. Despite being usually considered a solitary canid, a growing body of evidence, including the present work, is revealing how complex the long-range acoustic communication of maned wolves is and how we can use sequences of acoustic events to gain extensive information about cryptic species.

## Figures and Tables

**Table 1:** Maned wolves’ roar-bark sequences recorded passively at the Serra da Canastra National Park. At the top is the complete dataset (all recorders) and at the bottom the subsample of the 5 common recording sites used in all periods (only nights in which all 5 were active).

**All Recorders Periods:**	**Non-Reproductive**	**Mating**	**Gestation**	**Parturition**	**Initial Parental Care**
Nights recorded	37	53	82	57	31
Sequences	67	202	93	104	49
Interactive sequences	4	24	10	9	11
Average recorders	6.3 ± 1.2	12.4 ± 1.0	12.1 ± 1.2	11.8 ± 1.6	12 ± 0
Sequences by night by recorder	0.286 ± 0.522	0.303 ± 0.402	0.093 ± 0.120	0.154 ± 0.215	0.132 ± 0.362
Interactive sequences by night by recorder	0.014 ± 0.057	0.036 ± 0.087	0.011 ± 0.036	0.014 ± 0.044	0.030 ± 0.063
% of interactive sequences	2.4 ± 6.9%	14.8 ± 29.0%	10.5 ± 26.8%	14.1 ± 32.5%	33.1 ± 44.4%
Nights with sequences	16	38	44	32	16
% of nights with sequences	43.2%	71.7%	53.7%	56.1%	51.6%
Nights with interactive sequences	2	13	8	6	7
% of nights with interactive sequences	5.4%	24.5%	9.8%	10.5%	22.6%
**Five Recorders Periods:**	**Non-Reproductive**	**Mating**	**Gestation**	**Parturition**	**Initial Parental Care**
Nights recorded	21	50	69	59	21
Sequences	46	76	36	64	11
Interactive sequences	4	12	1	10	6
Sequences by night	2.190 ± 3.669	1.520 ± 2.801	0.522 ± 1.001	1.085 ± 3.218	0.524 ± 0.814
Interactive sequences by night	0.190 ± 0.602	0.240 ± 0.916	0.014 ± 0.120	0.169 ± 0.530	0.286 ± 0.644
% of interactive sequences	4.2 ± 10.4%	14.5 ± 31.5%	1.2 ± 5.5%	21.0 ± 38.8%	45.8 ± 50.2%
Nights with sequences	11	22	21	24	8
% of nights with sequences	52.4%	44.0%	30.4%	40.7%	38.1%
Nights with interactive sequences	2	6	1	4	4
% of nights with interactive sequences	9.5%	12.0%	1.4%	6.8%	19.0%

**Table 2:** Intervals of the linear mixed model of Total Duration of captive maned wolves roar-barks (*N* = 200). Values are shown in seconds. ‘Participants’ is the number of vocally interacting animals in the sequence from which the roar-bark was selected. There are 10 individuals, 6 females and 4 males. Total number of different sequences combined with individuals is 89.

Total Duration ~Sex + Participants + (1|Individual/Sequence)		Approximate 95% Confidence Intervals		
		Lower	Estimate	Upper
Intercept	(Female, one)	0.463	0.530	0.597
Fixed factors	Sex (males)	0.006	0.124	0.241
	Participants (two)	−0.023	0.008	0.038
	Participants (3more)	−0.011	0.017	0.044
Random factors	Individual	0.047	0.078	0.129
	Sequence in individual	1.69 × 10^−201^	1.78 × 10^−07^	1.87 × 10^+187^

## Data Availability

Raw tabulated data for the acoustic parameters is available in the Supplementary Materials. The audio files are available on request from the corresponding author. The data are not publicly available because the complete audio dataset is too large and not yet available in a public archive.

## Supplementary Materials

The following supporting information can be downloaded at: https://www.mdpi.com/article/10.3390/ani12091081/s1, Acoustic_parameters_dataset, Audio S1: interactive roar-bark sequence Casc_20140619_015530, Audio S2: interactive roar-bark sequence Bif_20160409_185414, Audio S3: interactive roar-bark sequence Rol_20160314_200004.
